# Personalized Decision-Making in Periodontal Therapy: Systemic and Demographic Factors Influencing Surgical vs. Non-Surgical Re-Treatment

**DOI:** 10.3390/jpm16060290

**Published:** 2026-05-27

**Authors:** Georgios S. Chatzopoulos, Larry F. Wolff

**Affiliations:** Department of Developmental and Surgical Sciences, Division of Periodontology, School of Dentistry, University of Minnesota, 515 Delaware Street SE, Minneapolis, MN 55455, USA; wolff001@umn.edu

**Keywords:** periodontal therapy, decision-making, surgical periodontal therapy, non-surgical periodontal therapy, retrospective cohort study, health disparities

## Abstract

**Background/Objectives**: The aim of this retrospective cohort study was to identify and analyze the demographic, systemic, and clinical factors associated with the type of periodontal therapy (surgical vs. non-surgical re-treatment) to better inform multifactorial approaches in periodontal treatment planning. **Methods**: Electronic health records were analyzed to identify two distinct patient cohorts. The surgical cohort included patients who received one or more surgical procedures (i.e., gingival flap, osseous surgery). The non-surgical re-treatment cohort included patients who received scaling and root planing on more than one occasion and had no history of surgical treatment. Bivariate analyses (*t*-tests and Chi-square tests) were used to compare cohort characteristics, and a multivariable binary logistic regression model was developed to identify independent predictors of receiving surgical treatment. **Results**: A total of 3794 patients were included in the final analysis. The non-surgical re-treatment cohort presented with significantly more severe periodontal disease at baseline, including a higher number of bleeding sites and deep pockets (*p* < 0.001). The logistic regression analysis revealed that a higher number of bleeding sites (OR = 0.984) and deep pockets (OR = 0.987) were significantly associated with a decreased likelihood of receiving surgical treatment. Patients with high blood pressure (OR = 0.620) or arthritis (OR = 0.611) also had significantly lower odds of receiving surgery. Conversely, White patients had significantly higher odds (OR = 1.366) of receiving surgical therapy compared to non-White patients. **Conclusions**: Within the limitations of this study, the selection between surgical and non-surgical periodontal therapy is a multifactorial decision. Initial disease severity, specific systemic conditions, and patient race were significant predictors of the treatment provided. These findings highlight the necessity of adopting a multifactorial risk assessment framework in periodontology—one that integrates systemic and demographic risk factors alongside clinical parameters to tailor equitable, patient-specific care.

## 1. Introduction

The management of periodontal disease is a complex process influenced by a variety of factors related to the patient and the professional environment [1]. Over the past few years, much has been learned about the relationship between risk factors—such as smoking, diabetes, and specific bacteria—and the progression of periodontal diseases [2,3]. This has led to a clinical landscape where practitioners often face the dilemma of whether to retain a compromised natural tooth through periodontal therapy or to extract and replace it, a key decision in contemporary treatment planning [4,5,6,7]. The ultimate goal remains the long-term maintenance of teeth in health, function, and comfort while meeting the esthetic expectations of the patient [8].

A cornerstone of modern periodontal practice is the detailed assessment of the “patient profile,” defined as the sum of all determinants and attributes that characterize an individual and their suitability for active therapy [1]. This profile extends beyond clinical measurements to include a patient’s demographics, socioeconomic background, general health, and dental history. This comprehensive evaluation is the cornerstone of personalized medicine in dentistry. By shifting away from a ‘one-size-fits-all’ approach focused solely on localized disease metrics, personalized periodontal medicine aims to customize healthcare decisions and practices to the individual patient. Systemic conditions, behavioral patterns, and lifestyle factors like smoking are critical components that not only influence disease susceptibility but also affect treatment decisions and outcomes [6]. A retrospective study by Chatzopoulos and Wolff confirmed the importance of these non-clinical variables, demonstrating that age, medical history, socioeconomic status, and insurance coverage were significantly associated with whether a patient received endodontic treatment or a dental implant [6].

In parallel with understanding the clinician’s perspective, there is a growing recognition of the importance of assessing patients’ preferences, a central tenet of Evidence-Based Dentistry [9]. Research suggests that aligning treatment with patient values can improve adherence, particularly in chronic conditions like periodontitis, where long-term patient engagement is crucial [10]. A recent scoping review highlighted that patients with periodontal disease prioritize avoiding tooth loss above all else, followed by treatment cost [9]. However, the same review concluded that the overall body of literature on patient preferences in periodontal therapy is scarce [9]. This reveals a significant gap in the literature; while we know that patient characteristics influence decisions in other areas of dentistry [11,12,13], and patient preferences are important, large-scale retrospective studies analyzing the combined influence of demographic, medical, and clinical profiles on the specific selection between surgical and repeated non-surgical periodontal pathways are lacking.

This study was designed to address this gap. Drawing from evidence that non-clinical factors influence dental treatment plans [13] and that a patient’s systemic and behavioral status should guide therapeutic objectives [1], it was hypothesized that a patient’s demographic characteristics, systemic medical conditions, and the initial severity of their periodontal disease are significantly associated with the type of periodontal therapy they receive (surgical versus non-surgical re-treatment). This hypothesis is built on the premise that the decision to pursue a more invasive surgical route—or a more conservative, repeated non-surgical approach—is a multifactorial decision based on the patient’s complete clinical and personal profile.

Therefore, the primary aim of this study was to utilize a large retrospective cohort to identify and analyze the demographic, systemic, and clinical factors associated with the type of periodontal therapy (surgical vs. non-surgical re-treatment) provided to patients with periodontitis. This approach mirrors the methodology used in similar dental epidemiology research [6]. By employing a multivariable logistic regression model, this study sought to determine which specific patient characteristics are statistically significant predictors of receiving surgical treatment over a course of repeated non-surgical interventions, thereby providing valuable insights into clinical practice patterns, building upon foundational studies that have clarified the indications and efficacy of both non-surgical and surgical modalities [14,15]. By identifying these key predictors, this study aims to contribute to the growing field of patient-centered treatment planning by providing evidence-based insights into how individual patient characteristics shape optimal therapeutic pathways.

## 2. Materials and Methods

### 2.1. Study Design and Data Source

This study was a retrospective cohort analysis utilizing de-identified patient data from the BigMouth Dental Data Repository. BigMouth is a large-scale, centralized, multi-institutional database encompassing electronic health records (EHRs) from several prominent academic dental institutions across the United States, representing a diverse, national patient pool. The dataset comprised information on patient demographics, self-reported medical history, and a comprehensive record of all dental procedures performed, identified by the American Dental Association’s Current Dental Terminology (CDT) codes. The datasets used and analyzed during the current study are available from the corresponding author. Clinical periodontal measurements recorded during patient examinations were also extracted. This study was conducted and reported in accordance with the Strengthening the Reporting of Observational Studies in Epidemiology (STROBE) guidelines for cohort studies. This retrospective study received a determination from the University of Minnesota Institutional Review Board (STUDY00016576) that it did not constitute research involving human subjects. Data for this study were extracted for patients receiving care between 2011 and 2022.

### 2.2. Cohort Definition

Patients included in the study were adults who had received periodontal therapy. To create distinct comparison groups, patients were assigned to one of two mutually exclusive cohorts based on the type of periodontal treatment they received.

- Surgical Cohort: This group included all patients who had received one or more surgical periodontal procedures. Assignment to this cohort was based on the presence of any of the following CDT codes in their treatment history: D4240 (Gingival Flap Procedure), D4241 (Gingival Flap Procedure, one to three teeth), D4245 (Apically Positioned Flap), D4260 (Osseous Surgery), D4261 (Osseous Surgery, one to three teeth), D4266 (Guided Tissue Regeneration), and D4274 (Distal/Mesial Wedge Procedure).
- Non-Surgical Re-treatment Cohort: This group included patients who had received non-surgical periodontal therapy on more than one occasion. Assignment was based on having a record of at least two procedures from the following CDT codes: D4341 (Periodontal Scaling and Root Planing, four or more teeth) and D4342 (Periodontal Scaling and Root Planing, one to three teeth). Importantly, periodontal maintenance procedures (CDT code D4910) were not utilized to define this cohort. This ensured the group reflected patients requiring repeated phases of active non-surgical intervention, rather than those undergoing routine, stable supportive periodontal therapy. Patients who had any record of a surgical periodontal procedure were explicitly excluded from this cohort to ensure the groups were distinct.

### 2.3. Variables

The primary outcome variable was the Treatment Group, a binary variable defined as Surgical or Non-Surgical Re-treatment.

The independent variables (predictors) were extracted from the EHR and categorized as follows:

- Demographic Characteristics: Age at baseline (continuous), gender (male/female), and self-reported race and ethnicity.
- Medical History and Lifestyle Factors: The presence or absence (‘Yes’/’No’) of the 10 most prevalent self-reported medical conditions was recorded. Smoking status (‘Yes’/’No’ to cigarette use) was also included. To ensure statistical viability in the regression model, we predefined the inclusion of the 10 conditions with the highest overall raw prevalence within the complete, unstratified dataset.
- Periodontal Status: Clinical measurements were taken from the patient’s first recorded “Initial Exam” to establish a baseline before the influence of extensive therapy. These variables included number of sites with bleeding on probing (BOP, continuous), number of periodontal pockets 4 mm or deeper (PPD ≥ 4 mm, continuous), mean probing depth (Mean PD, continuous), and mean clinical attachment level (Mean CAL, continuous).

### 2.4. Statistical Analysis

First, descriptive statistics were calculated for all variables. Continuous variables were summarized using means and standard deviations (SDs), while categorical variables were summarized using frequencies and percentages. Bivariate analyses were then performed to compare the characteristics of the two treatment cohorts. Independent samples *t*-tests were used for normally distributed continuous variables, while the non-parametric Mann–Whitney U test was utilized for highly skewed continuous variables (e.g., BOP sites, PPD ≥ 4 mm). Chi-square (χ^2^) tests were used for categorical variables. The bivariate comparisons presented were intended to be descriptive and exploratory in nature; therefore, no formal corrections for multiple testing were applied prior to the multivariable modeling.

To investigate the association between the independent variables and the likelihood of receiving surgical treatment, a multivariable binary logistic regression model was developed. The model included variables representing demographics, medical history, lifestyle, and baseline periodontal status. The results of the regression analysis are reported as odds ratios (ORs) with their corresponding 95% confidence intervals (CIs). This allowed for the identification of factors that were independently and significantly associated with the type of treatment a patient received.

For the multivariable binary logistic regression model, variable selection was guided by clinical relevance and the results of the bivariate analyses. All selected independent variables were entered into the model simultaneously (forced entry method). Prior to finalizing the model, multicollinearity among the independent variables was assessed using the Variance Inflation Factor (VIF); all VIF values were well below the standard threshold of 5, indicating no significant multicollinearity. The overall goodness-of-fit of the logistic regression model was evaluated using the Hosmer–Lemeshow test to ensure the model adequately fit the data.

Because this study utilized real-world electronic health record (EHR) data, missing values were present, predominantly in self-reported demographic categories. To address this, missing demographic variables (such as race and ethnicity) were categorized as ‘Unknown/Not Reported’ for the descriptive statistics to maintain the full cohort denominator. For the multivariable binary logistic regression model, a complete-case analysis approach was employed. Patients with missing values for any of the specific clinical, systemic, or demographic covariates included in the final model were excluded from that specific regression analysis.

A *p*-value of <0.05 was considered statistically significant for all tests.

## 3. Results

### 3.1. Patient Characteristics

**Figure 1:**
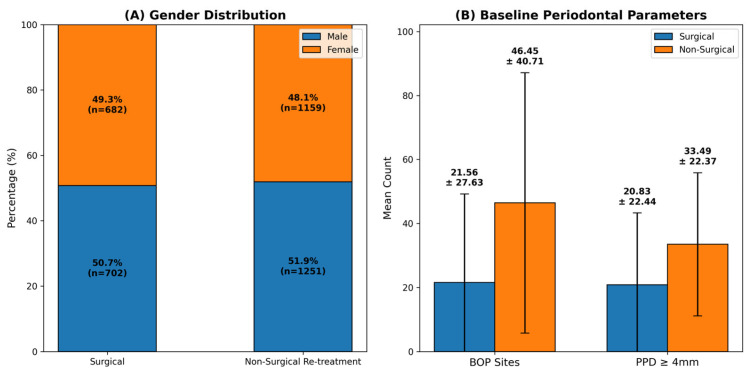
Baseline demographic and clinical characteristics of the study cohorts: (**A**) Stacked bar chart illustrating the gender distribution across the surgical (n = 1384) and non-surgical re-treatment (n = 2410) cohorts. (**B**) Clustered bar chart displaying the mean initial clinical parameters, including the number of bleeding on probing (BOP) sites and periodontal pockets (PPD) ≥ 4 mm, highlighting the higher baseline disease burden in the non-surgical group.

A total of 3794 patients met the final inclusion criteria for the analysis. There were 1384 patients in the surgical treatment cohort and 2410 patients in the non-surgical re-treatment cohort. Table 1 provides a comprehensive comparison of the demographic, medical, and clinical characteristics of these two groups. Furthermore, the baseline periodontal parameters (bleeding on probing and deep pockets) and gender distributions are visually summarized in Figure 1.

### 3.2. Bivariate Analysis

Bivariate analysis revealed no statistically significant difference in the mean baseline age or gender distribution between the cohorts. However, significant differences were observed in racial and ethnic composition. The surgical cohort comprised a significantly higher proportion of White patients (55.2% vs. 45.9%, *p* < 0.001). Regarding ethnicity, the non-surgical group had a higher proportion of Hispanic patients (12.3%) compared to the surgical group (7.7%). These baseline differences underscore that the two groups represent distinct real-world clinical subpopulations rather than equivalent cohorts.

### 3.3. Multivariable Logistic Regression

The logistic regression model (Table 2) revealed that the strongest predictors of the treatment path were the patient’s baseline periodontal status and specific medical conditions.

Periodontal Status: After adjusting for all other factors, a higher number of sites with bleeding on probing (OR = 0.984, *p* < 0.001) and a greater number of deep pockets (OR = 0.987, *p* < 0.001) were both significantly associated with a decreased likelihood of receiving surgical treatment.

Systemic Health: Patients with a history of high blood pressure (OR = 0.620, *p* = 0.007) or arthritis (OR = 0.611, *p* = 0.010) had significantly lower odds of being in the surgical group.

Demographics: White patients had significantly higher odds (OR = 1.366, *p* = 0.035) of receiving surgical treatment compared to non-White patients.

## 4. Discussion

This study provides a comprehensive analysis of the factors influencing the selection between surgical and non-surgical re-treatment for periodontal disease, confirming the hypothesis that the decision is multifactorial [1]. The logistic regression analysis revealed that the initial severity of periodontal disease, specific systemic health conditions, and patient demographics are significant predictors of the treatment pathway, a finding that aligns with similar research in other areas of dental decision-making [6]. One of the most compelling findings was that patients presenting with more severe and widespread periodontal disease, characterized by a higher number of bleeding sites and deep pockets, were significantly more likely to be managed with repeated non-surgical therapy. This counterintuitive finding suggests that in this university setting, clinicians may opt for conservative stabilization (repeated scaling) rather than invasive surgery when the disease burden is high, and the prognosis for surgical success may be guarded.

This counterintuitive finding must be interpreted with caution, as it is highly likely that it reflects selection bias inherent in the retrospective design rather than a standardized clinical practice of avoiding surgery in severe cases. While it is possible that clinicians opted for conservative, non-surgical management as a stabilizing or definitive measure for patients presenting with numerous teeth of questionable or poor prognosis, several alternative explanations must be considered. For instance, patients presenting with severe, generalized disease may also face more significant financial or socioeconomic barriers that limit their ability to accept comprehensive, higher-cost surgical treatment plans, thus restricting them to repeated non-surgical care. Furthermore, patients with severe disease at baseline might have a history of poor oral hygiene compliance or irregular dental attendance, prompting clinicians to withhold advanced surgical interventions until adequate plaque control and behavioral compliance are demonstrated. Therefore, the association between higher baseline disease severity and non-surgical re-treatment is likely confounded by these unmeasured patient-level variables, rather than reflecting true clinical efficacy or preference.

The influence of systemic health and demographics on treatment selection is consistent with previous research that highlights the role of non-clinical factors in dental decision-making [6,12,13]. The finding that patients with high blood pressure or arthritis were less likely to receive surgical treatment may indicate a clinical tendency to avoid invasive procedures in medically compromised individuals. This aligns with the broader principle of tailoring treatment to a patient’s overall health profile [1]. A notable demographic finding was that White patients were more likely to receive surgical treatment compared to non-White patients. However, this observation must be interpreted with considerable caution. Because this analysis did not include granular data on socioeconomic status, specific dental insurance coverage (e.g., Medicaid versus private PPO), or geographic access to specialized care, it is highly probable that race is acting as a proxy for these unmeasured variables. Socioeconomic barriers and the availability of healthcare resources are known to profoundly influence a patient’s ability to accept and afford comprehensive surgical treatment plans. Therefore, rather than indicating a direct racial disparity in clinical decision-making, this finding underscores the critical need for future models to incorporate comprehensive socioeconomic indicators to truly understand the drivers of treatment acceptance and access.

When interpreting these results, it is crucial to distinguish between statistical and clinical significance. Because this study utilized a large cohort of 3794 patients, the statistical power to detect minor differences was exceptionally high. For instance, while a higher number of bleeding sites (OR = 0.984) and deep pockets (OR = 0.987) were highly statistically significant (*p* < 0.001) predictors of receiving non-surgical therapy, their odds ratios are extremely close to 1. This means that a single-unit increase—having just one more bleeding site or one more deep pocket—only alters the odds of receiving surgical treatment by approximately 1.3% to 1.6%. Therefore, while the overall disease burden is a statistically valid predictor of the treatment pathway, the clinical significance of minor unit variations in BOP or PPD is negligible when compared to the much larger impact of binary systemic factors, such as a diagnosis of high blood pressure (OR = 0.620) or arthritis (OR = 0.611).

This study is not without its limitations. A primary limitation of this retrospective cohort study is the inherent presence of confounding by indication. Because treatment allocation was not randomized, the clinical decision to pursue surgical versus repeated non-surgical therapy was inherently entangled with the patient’s baseline presentation and various unmeasured factors. Specifically, our analysis was unable to control for several crucial variables that heavily influence clinical decision-making, including individual tooth prognosis, specific patient preferences, clinician experience and treatment philosophy, precise socioeconomic status, and detailed insurance coverage. Consequently, the findings presented in this study must be interpreted strictly as associations observed within a real-world clinical setting, rather than as causal determinants of therapy choice.

Furthermore, it must be acknowledged that the two cohorts were not perfectly comparable at baseline, introducing a degree of selection bias. The interplay between systemic comorbidities and periodontal severity likely plays a significant role in this treatment allocation. Conditions such as diabetes and cardiovascular disease are known to exacerbate systemic inflammation, often resulting in a more rapid and severe progression of periodontal tissue loss. Consequently, medically compromised patients frequently present with a higher baseline disease burden. However, these same systemic factors concurrently increase the risks associated with invasive surgical procedures and impair wound healing. The criteria used to define the non-surgical re-treatment group inherently capture a heterogeneous patient population. This group likely includes not only patients whose disease was successfully stabilized with conservative therapy but also those who were deemed poor surgical candidates due to systemic comorbidities, had teeth with hopeless prognoses, faced financial constraints, or simply refused invasive surgical recommendations. Therefore, the observation that severe disease frequently results in a non-surgical pathway may be largely driven by clinicians appropriately electing conservative, repeated mechanical debridement for patients whose systemic health precludes surgical intervention, despite their advanced periodontal destruction. Consequently, our comparative analyses highlight real-world treatment allocation patterns rather than outcomes between perfectly matched populations.

Furthermore, the data are from a single university setting, which may not be generalizable to private practice or other healthcare systems [6]. The self-reported nature of medical histories also carries a potential for recall bias or inaccuracies, although it is considered a reliable method in many studies [16,17]. Additionally, the EHR dataset limited the assessment of smoking to a binary variable (Yes/No). The inability to quantify smoking intensity (pack-years) or differentiate between current and former smokers likely blunted the statistical sensitivity needed to detect the true influence of tobacco use on treatment planning within this model.

Despite these limitations, the findings of this study have significant clinical implications. They underscore that the management of periodontitis is not a one-size-fits-all endeavor and that patient-level factors beyond clinical measurements play a crucial role in the real-world application of therapeutic guidelines [1]. The identified associations, particularly the racial disparity in the provision of surgical care, highlight a critical area for further investigation and intervention to ensure equitable care. Future prospective studies should aim to incorporate patient-reported outcomes and preferences, as well as detailed socioeconomic data, to build a more complete picture of the decision-making process in periodontal therapy. Understanding these complex interactions is essential for optimizing treatment outcomes and promoting patient-centered care.

## 5. Conclusions

This study demonstrates that the therapeutic path for patients with periodontitis is a multifactorial process, reflecting complex real-world treatment patterns rather than standardized clinical algorithms. The initial severity of periodontal disease, the presence of comorbidities such as high blood pressure and arthritis, and the patient’s race were all identified as significant predictors of receiving surgical versus non-surgical re-treatment. The finding that more severe initial disease predicted a non-surgical course likely reflects the heterogeneous nature of this cohort—which may include medically compromised individuals or those refusing surgery—and may be influenced by unmeasured socioeconomic and patient-level factors. This highlights the complexity of real-world treatment allocation and the need for further investigation incorporating detailed socioeconomic status and healthcare access variables to fully understand these dynamics and ensure equitable care delivery.

Ultimately, this research contributes to our understanding of clinical decision-making by demonstrating that the management of periodontitis extends beyond standardizing care based solely on clinical pocket depths. The observed associations underscore that a patient’s systemic health profile and demographic background play a significant role in real-world treatment allocation. While this study highlights existing practice patterns rather than active individualized decision-making, it reinforces the necessity of adopting a comprehensive, patient-centered approach to treatment planning—one that integrates clinical, systemic, and demographic variables to ensure care is both clinically sound and equitably delivered.

## Figures and Tables

**Table 1:** Complete patient profile comparison: surgical vs. non-surgical re-treatment.

Category	Characteristic	Surgical Cohort	Non-Surgical Re-treatment Cohort	*p*-Value
Demographics	Age	57.49 ± 13.17	57.00 ± 14.34	0.281
	Gender	M: 702, F: 682	M: 1251, F: 1159	0.248
	Ethnicity			
	Hispanic	106 (7.7%)	298 (12.3%)	<0.001
	Non-Hispanic	871 (62.9%)	1130 (46.8%)	<0.001
	Others	118 (8.5%)	86 (3.6%)	<0.001
	Unknown/Not reported	289	896	
	Race			
	White	764 (55.2%)	1109 (45.9%)	<0.001
	Black/African American	99 (7.2%)	309 (12.8%)	<0.001
	Asian	93 (6.7%)	134 (5.6%)	0.164
	Pacific Islander	17 (1.2%)	12 (0.5%)	0.022
	American Indian/Alaskan Native	7 (0.5%)	5 (0.2%)	0.201
	Some Other Race	195 (14.1%)	127 (5.3%)	<0.001
	Unknown/Not reported	209	714	
Lifestyle Factors	Smoking	179 (12.9%)	354 (14.7%)	<0.001
	Alcohol consumption	506 (36.6%)	796 (33.0%)	<0.001
Top 10 Medical Conditions	Hypertension	426 (30.8%)	886 (36.7%)	0.003
	Cardiovascular diseases	440 (31.8%)	632 (26.2%)	0.057
	Arthritis	218 (15.8%)	445 (18.4%)	0.004
	Diabetes	168 (12.1%)	345 (14.3%)	0.092
	Anxiety	158 (11.4%)	343 (14.2%)	0.038
	Depression	141 (10.2%)	275 (11.4%)	0.279
	Thyroid disorder	129 (9.3%)	290 (12.0%)	0.016
	Gastrointestinal problems	126 (9.1%)	240 (9.9%)	0.449
	Stroke	95 (6.9%)	134 (5.6%)	0.106
	Anemia	89 (6.4%)	185 (7.7%)	0.155
Top 10 Current Medications	Aspirin (acetyl salicylic acid—ASA)	134 (9.7%)	206 (8.5%)	0.239
	Lisinopril	75 (6.0%)	147 (6.7%)	0.488
	Metformin	59 (4.7%)	103 (4.7%)	1.000
	Omeprazole	41 (3.3%)	67 (3.0%)	0.771
	Simvastatin	49 (3.9%)	81 (3.7%)	0.784
	Multivitamins	64 (5.1%)	61 (2.8%)	0.001
	Amlodipine	39 (3.1%)	99 (4.5%)	0.060
	Metoprolol	42 (3.4%)	73 (3.3%)	1.000
	Calcium	56 (4.5%)	46 (2.1%)	<0.001
Periodontal Status (Initial Exam)	Mean Probing Depth (PD)	2.74 ± 0.72	2.99 ± 0.54	<0.001
	Mean Clinical Attachment Loss (CAL)	2.63 ± 0.95	2.56 ± 1.18	0.157
	Bleeding on probing (BOP) sites	21.56 ± 27.63	46.45 ± 40.71	<0.001
	PD Greater or Equal 4	20.83 ± 22.44	33.49 ± 22.37	<0.001

*p*-values calculated using the independent samples *t*-test for normally distributed continuous variables (Age, Mean PD, Mean CAL), the Mann–Whitney U test for skewed continuous variables (BOP sites, PPD ≥ 4 mm), and the Pearson Chi-square test for all categorical variables.

**Table 2:** Summary of logistic regression results.

Characteristic	Odds Ratio	95% CI	*p*-Value
Demographics			
Age	1.006	[0.994, 1.017]	0.336
Gender (Female)	1.086	[0.826, 1.427]	0.554
Ethnicity: Hispanic	0.665	[0.437, 1.014]	0.058
Race: White	1.366	[1.023, 1.823]	0.035
Lifestyle Factors			
Smoker (Cigarettes)	0.807	[0.465, 1.402]	0.447
Medical Conditions			
High Blood Pressure	0.620	[0.436, 0.880]	0.007
Arthritis	0.611	[0.420, 0.888]	0.010
Cardiovascular/Heart Problem	1.497	[0.935, 2.395]	0.093
Diabetes	1.133	[0.706, 1.819]	0.605
Periodontal Status			
BOP number of sites	0.984	[0.979, 0.989]	<0.001
PPD ≥ 4 mm	0.987	[0.980, 0.994]	<0.001

Odds ratios (ORs) for categorical variables are calculated against the following reference categories: Male (for Gender), Non-White (for Race), Non-Hispanic (for Ethnicity), and ‘No’ or ‘Absence of condition’ (for Smoking status and all medical conditions).

## Data Availability

The datasets used and/or analyzed during the current study are available from the corresponding author.
